# Delphinidin and Its Glycosides’ War on Cancer: Preclinical Perspectives

**DOI:** 10.3390/ijms222111500

**Published:** 2021-10-25

**Authors:** Anshul Sharma, Hyo-Kyoung Choi, Yeon-Kye Kim, Hae-Jeung Lee

**Affiliations:** 1 Department of Food and Nutrition, College of Bionanotechnology, Gachon University, Seongnam-si 13120, Gyeonggi-do, Korea; anshul.silb18@gmail.com; 2 Korea Food Research Institute, Wanju-gun 55365, Jeollabuk-do, Korea; chkyoung@kfri.re.kr; 3 Food Safety and Processing Research Division, National Institute of Fisheries Science, Gijang-eup, Busan 46083, Korea; yeonkyekim@korea.kr; 4 Institute for Aging and Clinical Nutrition Research, Gachon University, Seongnam-si 13120, Gyeonggi-do, Korea

**Keywords:** delphinidin, cancer, proliferation, metastasis, anthocyanidins, anthocyanins

## Abstract

Until now, several studies have looked at the issue of anthocyanin and cancer, namely the preventive and inhibitory effects of anthocyanins, as well as the underlying molecular processes. However, no targeted review is available regarding the anticarcinogenic effects of delphinidin and its glycosides on various cancers and their plausible molecular mechanisms. Considerable evidence shows significant anticancer properties of delphinidin-rich preparations and delphinidin alone both in vitro and in vivo. This review covers the in vitro and preclinical implications of delphinidin-mediated cell protection and cancer prevention; thus, we strongly recommend that delphinidin-rich preparations be further investigated as potential functional food, dietary antioxidant supplements, and natural health products targeting specific chronic diseases, including cancer. In addition to in vitro investigations, future research should focus on more animal and human studies to determine the true potential of delphinidin.

## 1. Introduction

Plants have evolved their genomes over time and produced a vast array of specialized molecules, known as secondary metabolites, to cope with biotic and abiotic stresses. Humans use a number of these metabolites as anticancer medicines, antioxidants, enzyme inhibitors, immunomodulators, antibiotics, and plant as well as animal growth promoters to improve their health. They are produced by biosynthetic processes that use primary metabolites and intermediates. These are primarily produced in the cytosol (anthocyanins), chloroplasts (terpenoids), or mitochondria (amines), but are stored in the vacuole for use when needed. Anthocyanins (of the Greek anthos = flower and kianos = blue), a subfamily of flavonoids, are the most abundant water-soluble pigments found in plants [1]. Anthocyanins have tremendous economic value as medications and dietary supplements (and have desirable coloring properties) without any side effects [2]. Anthocyanins provide many health benefits that have been inferred by scientists based on their antioxidant and anti-inflammatory properties [3]. New research efforts and their successes on anthocyanins and functional food will contribute to more sustainable agriculture, health, and environments. Anthocyanin aglycones are known as anthocyanidins. There are roughly 23 different varieties of anthocyanidins, with cyanidin, delphinidin, pelargonidin, malvidin, peonidin, and petunidin being the most frequent [4]. Delphinidin (2-(3,4,5-trihydroxyphenyl) chromenylium-3,5,7-triol) is one of the most valuable polyphenol anthocyanidin classes and possesses significant biological activity. It is one of the most common pigmented anthocyanidins, found in a wide variety of brightly colored fruits and vegetables (e.g., grapes, berries, sweet potatoes, and pigmented cabbages) and dietary supplements [5]. Secondary structural changes aid in delphinidin’s stability, bioavailability, and metamorphosis. The glycosylation of delphinidin, for example, has been related to improved stability, which is altered in vivo by physicochemical and biological variables [6]. Delphinidin possesses many health-promoting activities, including antioxidant, anti-inflammatory, hepatoprotective, antidiabetic, antimicrobial, neuroprotection, anti-adipogenesis, cardiovascular protection, and anticancer effects [6,7].

Cancer progress implicates initiation, progression, invasion, and metastasis. Globocan in 2018 predicted that the number of cancer cases and cancer deaths globally will rise by 63.1% and 71.1%, respectively, by 2040 [8,9]. Hereditary mutations are thought to be responsible for 5–10% of all malignancies, while environmental variables and lifestyle choices are responsible for the vast majority (90–95%) [10]. Alcohol use, physical activity, cigarette smoking, nutrition, and anthropometry are examples of lifestyle factors that have been linked to cancer risk [10,11,12]. Increasing evidence has unveiled the fact that moderate oxidative stress and persistent inflammation in tumor microenvironments have been linked to cancer development. A huge volume of literature is available discussing the cancer-preventive and cancer-managing capabilities of anthocyanins [2,13,14]. Although a recent study [6] outlines the multifaceted benefits of delphinidin, a complete assessment on the precise understanding of its anticancer effects is inadequate, necessitating the investigation of its molecular mechanisms and targets for the treatment and management of malignancies.

Thus, the present review highlights significant findings regarding the delphinidin-mediated suppression of various cancers as well as the underlying mechanisms. The review concludes with an outlook on the exciting future possibilities and scientific challenges for the use of delphinidin in cancer prevention/treatment. Although anthocyanins have been used in in vitro and in vivo animal research, and compelling evidence for the regulation of several signaling pathways has been presented, much work remains to be done to bring biomarker endpoints closer to human-use trials in the clinic.

## 2. Structure

**Figure 1:**
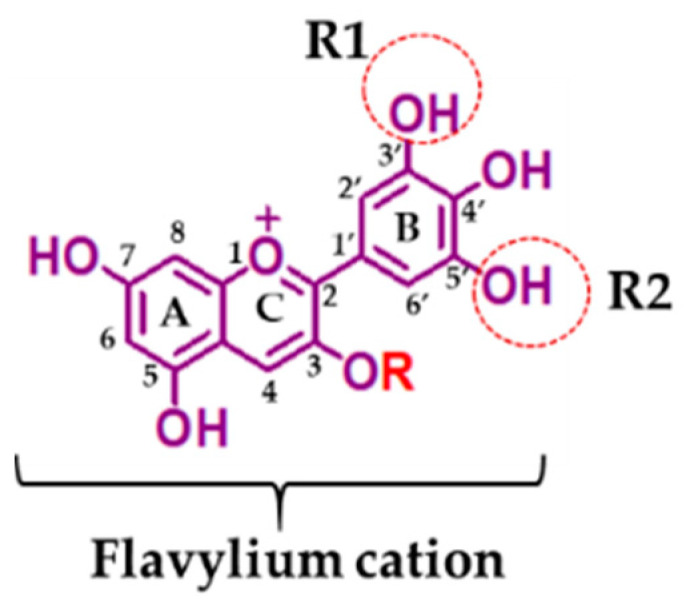
Chemical representation of delphinidin and its glycosides. R = H (delphinidin), R = glucoside (delphinidin-3-*O*-glucoside), R = galactoside (delphinidin-3-*O*-galactoside), R = rutinoside (delphinidin-3-*O*-rutinoside), R = arabinoside, (delphinidin-3-*O*-arabinoside), and R = sambubioside (delphinidin-3-*O*-sambubioside).

Anthocyanins most commonly present a trisaccharide, disaccharide, or monosaccharide unit. Hydrolyzed anthocyanins yield anthocyanidins and sugars [15]. As a result, anthocyanidin or anthocyanin aglycones have no sugar moiety attached to the molecular structure of the flavylium cation, which is made up of two benzoyl rings (A and B) separated by a heterocyclic ring (C) (Figure 1) [16]. The presence of a substitute group such as hydroxide (OH), a hydrogen (H) atom, or methoxy at positions R1 and R2 differentiated them into different types. Delphinidin is an anthocyanidin represented by the flavylium cation holding OH substitutes at both R1 and R2 positions. Delphinidin-based anthocyanins are transformed into 2, 4, 6-trihydroxybenzaldehyde, gallic, and syringic acids [14,17].

## 3. Bioavailability Studies

As we know, anthocyanins, largely consumed by humans, are related to a decreased risk of developing cancer and other metabolic disorders. However, their beneficial properties strongly depend on their bioavailability. The amount of polyphenol that is routinely digested, absorbed, and metabolized is referred to as bioavailability. A recent study on bioavailability using maqui berry (*Aristotelia chilensis*) extract (MBE) powder (trade name Delphinol^®^), the maximum concentration of delphinidin-3-*O*-glucoside (D3G) could reach approximately 0.64 μg/mL in healthy humans. Pharmacokinetic studies show that the value of the area under the plasma concentration–time curve (AUC) from 0 h to 8 h is 84.9 nmol/L·h and the value of C_max_ levels varies from 21.39 to 63.55 nmol/L. The maximum D3G concentration was noted at 1.0 ± 0.3 h [18].

From another study of anthocyanins on human subjects, the reported bioavailabilities were delphinidin-3-*O*-galactoside (0.48%), D3G (0.14%), and delphinidin-3-*O*-arabinoside (0.14%). The maximum concentrations of delphinidin were found 2 h after consumption, while the glucuronide conjugates of delphinidin (phase II metabolites) were found 6.3 h later [19].

## 4. Delphinidin and Anticancer Activities

Biologically active phytoconstituents such as anthocyanins and anthocyanidins have shown a variety of diverse and sometimes additive mechanisms for preventing cancer. The prospective mechanisms of delphinidin and its glycosides include the induction of apoptosis and autophagy, the suppression of cell migration, anti-proliferation, anti-angiogenesis, the modulation of signaling pathways, and concentration-dependent decrease and increase in the number of cells in the G0/G1 and G2/M phases, respectively [20,21,22]. Anthocyanidins may decrease cancer cell proliferation by targeting receptor tyrosine kinases (RTKs), examples of which include epidermal growth factor receptor (EGFR), platelet-derived growth factor receptor (PDGFR), and vascular endothelial growth factor (VEGF)/VEGF receptor (VEGFR) and modulating Ras/mitogen-activated protein kinase (MAPK) and phosphatidylinositol-3-kinase (PI3K)/Akt signaling pathways [23,24]. Suppression of the Wnt/β-catenin signaling pathway (also known as the canonical Wnt signaling pathway) by delphinidin is also considered as another approach for evaluating its cancer-preventive and therapeutic effects [25]. Delphinidin may also prevent cancer by altering the expression of phase II antioxidant enzymes to achieve antioxidation via the nuclear-factor-E2-related factor 2/antioxidant response element (Nrf2/ARE) signaling system [26], as well as suppress inflammation by working on the PI3K/Akt and nuclear factor-kappa B (NF-κB) pathways to lower cyclooxygenase-2 (COX-2) and induce nitric oxide synthase (iNOS) synthesis. Delphinidin reduces RTK activity and targets the MAPK pathway as well as the activator protein 1 (AP-1) factor, which may help to avoid malignant transformation during the beginning of cancer [27].

**Figure 2:**
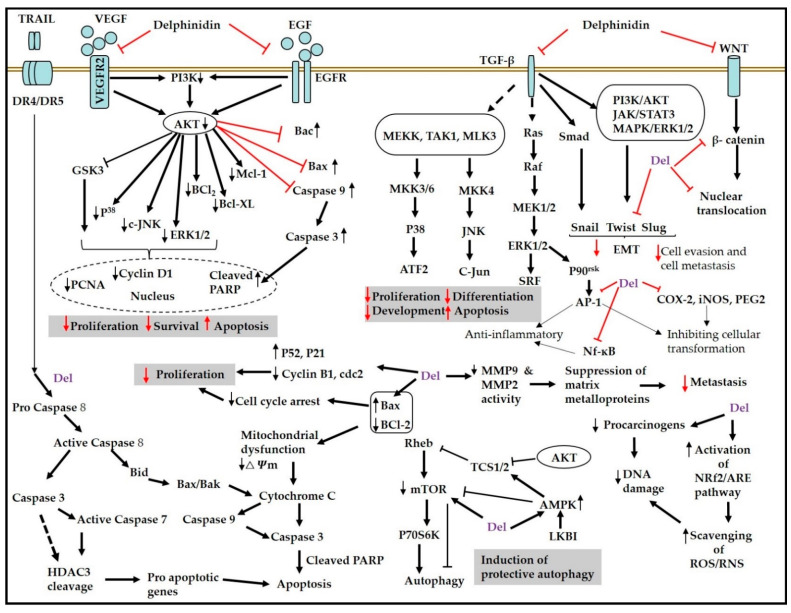
Plausible chemotherapeutic and chemopreventive working mechanisms of delphinidin and its glycosides [14,20,21,22,24,25,26,28,29,30,31,32].

Anthocyanins can induce the expression of *p53*, *p21*, and *p27* genes, whose products can combine with multiple-cyclin cyclin dependent kinases (CDKs) to down-regulate the expression of CDK-1 and CDK-2, as well as inhibit the expression of cyclin A, cyclin B, cyclin D, and cyclin E, all of which promote the expression of CDK inhibitors and cause the death of cancer cells. Anthocyanins activate caspases, which are mediated via reactive oxygen species (ROSs) and c-Jun N-terminal kinase (JNK)/p38-MAPK, causing cancer cells to die. By blocking the VEGF signaling pathway and reducing extracellular matrix breakdown via matrix metalloproteinases-2/9 (MMP-2/-9), anthocyanins may have anti-metastatic effects [22]. Figure 2 summarizes the features of the working mechanisms and therapeutic as well as preventive effects of delphinidin and its glycosides on various cancer cells.

### 4.1. Effect of Delphinidin against Prostate Cancer

NF-κB is a crucial cell process regulator that controls inflammation, proliferation, and apoptosis [33]. It has been shown to promote resistance to apoptosis induced via a range of chemotherapeutic agents, and it is currently being researched as an active target. Hafeez et al. [34] observed that delphinidin suppressed cell growth arrest and triggered caspase-dependent death in prostate cancer cells (PC-3) in vitro in a dose-dependent manner by lowering phosphorylations of IκB kinase γ, NF-κB inhibitory protein IκB-α, and restricting NF-κB DNA binding activity. The induction of apoptosis by delphinidin was facilitated by caspase activation, as evidenced by the fact that the degree of apoptosis was significantly reduced when an inhibitor (N-benzyloxycarbonyl-ValAla-Asp (OMe)-fluromethylketone) was present. Furthermore, delphinidin treatment significantly reduced tumor formation and NF-κB protein levels in mice with prostate cancer tumors in vivo. The animals were not harmed by the delphinidin doses since they did not gain weight or reduce their amount of food. After 12 weeks of assessments, the differences between the tumors of the two groups (control and treated) were considerable, demonstrating an anti-angiogenic effect on tumor cells (Table 1). In addition to diverse physiological processes, including embryogenesis, the Wnt/β-catenin signaling pathway is linked to tissue homeostasis and cancer in adults [35]. There is conclusive evidence that dysregulation of the Wnt/β-catenin signaling pathway has a role in the development and progression of a wide range of cancers [36,37]. Hence, suppression of the Wnt/β-catenin signaling pathway is another potential strategy for cancer-preventive and therapeutic agents. Delphinidin has been shown to downregulate the expression of β-catenin, its downstream target protein expression, and inhibit its nuclear translocation. Expressions of the proteins of the destruction complex of β-catenin were enhanced. Delphinidin suppressed the growth of PC-3 cells by blocking the Wnt/β-catenin signaling pathway (Table 1) [25]. More research on inhibitors, activators, and antagonists of the Wnt/β-catenin signaling pathway is needed to rationalize novel and possible cancer therapy options.

Another cancer treatment strategy is to combine tumor-necrosis-factor-related apoptosis-inducing ligands (TRAILs) and bioactive components to increase apoptosis in TRAIL-resistant tumor cells. In vitro and in vivo, the interaction of TRAILs, such as TRAILR1 (also known as DR4) and TRAILR2 (also known as DR5), with death receptors (DRs) on the surface of cancer cells can cause apoptotic cell death signaling via the death-receptor-mediated apoptosis pathway, with little effect on normal cells [38]. As a result, researchers have begun to look for biotherapeutic compounds that may activate TRAILs. Ko and coworkers have shown that delphinidin could resensitize TRAIL-resistant human prostate cancer cells and activate caspase activation pathways for the first time. The researchers determined that combining delphinidin with TRAIL generated caspase-dependent apoptosis since apoptosis was stopped after treatment with an inhibitor (zVAD), resulting in decreased levels of caspase-8, caspase-9, cleaved caspase-3, and caspase-7 (Table 1). Apoptosis was induced by cleaving histone deacetylase 3 (HDAC3) and activating DR5 expression [32]. HDACs have been linked to the deacetylation of p53, NF-κB, and signal transducer and activator of transcription (s) (STATs), which regulate the expression of several genes. In human prostate cancer LNCaP (a wild-type p53) cells, delphinidin causes caspase-mediated apoptosis, leading to HDAC3 inactivation and cleavage, by increasing caspase-3, caspase-7, and caspase-8 activity and by activating and stabilizing p53 in a dose- and time-dependent manner. These findings reveal that delphinidin causes p53-mediated apoptosis in LNCaP cells by decreasing HDAC activity and promoting p53 acetylation. As a result, delphinidin could be beneficial in preventing prostate cancer [29]. In another study, delphinidin-3-glucoside exhibits inhibitory effects on cell growth induced by dihydrotestosterone (DHT) in LNCaP cells. This might be associated with the abrogation of the nuclear accumulation of the androgen receptor (AR) and the reduced expressions of steroid 5α-reductase type 1 and prostate-specific antigen, induced by DHT [39].

### 4.2. Effect of Delphinidin against Ovarian Cancer

The binding and subsequent activation of tropomyosin-related kinase B (TrkB), a ty-rosine kinase receptor, by a brain-derived neurotrophic factor (BDNF) is crucial for nervous system development [40]. On the other hand, BDNF increases tumor cell growth and metastasis via tyrosine kinase receptors via numerous pathways, such as Akt, MAPK, and the mammalian target of rapamycin (mTOR) in cancer [41]. Delphinidin has been found to inhibit SKOV3 ovarian cancer cell metastasis by blocking BDNF-mediated invasion and migration as well as the production of BDNF-mediated downstream factors, including MMP-2/-9. In SKOV3 ovarian cancer cells treated with BDNF, delphinidin reduced Akt activation and NF-κB nuclear translocation. The study described the anti-metastatic effect of delphinidin on human ovarian cancer cells (Table 1) [42].

Various chemicals or medications that block the PI3K/Akt signaling pathway have been shown to treat a range of cancers [43]. Akt activity was inhibited by delphinidin treatment, underlining that this anthocyanidin strongly affects ovarian cancer [22,44]. Lim and Song [22] established that delphinidin exhibits antiproliferative effects on SKOV3 human ovarian cancer cells. They observed that delphinidin inhibited Akt, ribosomal protein S (S6), and ribosomal protein S6 kinase β-1 (P70S6K), PI3K downstream proteins, by lowering their phosphorylation. Furthermore, phosphorylated MAPK signaling proteins such as extracellular signal-regulated kinase (ERK)1/2 and P38 were downregulated with increasing doses of delphinidin, although JNK phosphorylation was unaltered. Delphinidin increased the rate of apoptosis in SKOV3 cells by inducing DNA fragmentation. The study describes the synergistic effect of delphinidin with paclitaxel (an anticancer drug) in SKOV3 cells. In a similar line, another study found that delphinidin had a more significant inhibitory effect on ES2 (representative of ovarian clear-cell carcinoma) cells in the presence of pharmacological inhibitors of signaling pathways, including LY294002 (PI3K inhibitor), U0126 (ERK1/2 MAPK inhibitor), and SB203580 (p38 MAPK inhibitor). Delphinidin suppressed phosphorylation of PI3K/Akt and ERK1/2/JNK, resulting in antiproliferative and anti-metastatic activities (Table 1) [44]. Collectively, these findings show that delphinidin may play a chemotherapeutic role in ovarian cancer cell prevention and progression by inactivating PI3K/Akt and ERK1/2 MAPK signaling pathways. The role of delphinidin in sensitizing PEO1 and SKOV3 cells has recently been demonstrated. PEO1 cells were harmed more when delphinidin and 3-bromopyruvic acid (an anticancer agent) were mixed. On the other hand, SKOV3 was less affected by the combination [1].

### 4.3. Effect of Delphinidin against Colorectal Cancer

Phosphoglycerate kinase 1 (PGK1) is a crucial ATP-producing enzyme in the glycolytic pathway, which is regulated by the hypoxia-inducible transcriptional factor (HIF-1) and plays a role in cancer progression and development [45]. In studies on the human colon cancer HT-29 cell line, several researchers discovered PGK1 as a possible biomarker of intracellular oxidative damage. In cells exposed to 50 µM H_2_O_2_ for 24 h, PGK1 expression was considerably elevated. PGK1 levels beyond a certain threshold are connected to tumor survival and angiogenesis. Surprisingly, delphinidin-treated cells had decreased quantities of this protein. The antioxidant capabilities of delphinidin, according to these experts, may aid in anticancer effects [46]. Agents capable of triggering apoptosis in cancer cells are now generally recognized as having the potential to develop mechanism-based cancer prevention and treatment. One study described how delphinidin treatment triggered caspase activation and PARP cleavage, two legitimate apoptotic pathways in HCT-116 cells. Delphinidin increased the expression of B-cell lymphoma 2 (Bcl-2)-associated X protein (BAX, a proapoptotic protein) while decreasing the expression of Bcl-2 (an antiapoptotic protein), causing cell cycle arrest by inhibiting the G2/M phase. Delphinidin boosted the tumor suppressor protein p53 as well as its downstream target p21. Both cyclin B1 and cell division cycle 2 kinase (cdc2) expression were dose-dependently reduced. The study discovered that treating HCT-116 cells with delphinidin-induced apoptosis and downregulation of the NF-κB pathway, as evidenced by the reduced IκB phosphorylation, lowered p65 subunit expression and suppressed NF-κB nuclear translocation (Table 1) [47]. Considering the molecular mechanisms responsible for this anticancer behavior, another recent study found that delphinidin therapy inhibited the phosphorylation of STAT-3 and MAPKinase signaling pathways, as well as the production of proapoptotic protein, causing HCT-116 cells to apoptosis. Delphinidin also reduced antioxidant levels, generated ROS levels, and resulted in the loss of mitochondrial membrane potential (ΔΨm) [48]. The results are in accordance with the results obtained by Yun et al. [47], showing the antiapoptotic potential of delphinidin in HCT-116 cells.

Anthocyanins have been demonstrated in several trials to help prevent the formation of colorectal cancer via improving microRNA (miRNA) regulation [49]. In this line, delphinidin was considered to prevent colorectal (DLD-1, SW480, and SW620) cancer cells from invading and migrating without changing their proliferative state. Delphinidin lowers integrin levels and promotes focal adhesion kinase (FAK) signaling while raising miR-204-3p (miRNA) expression. Delphinidin’s effects on cell invasion, integrin levels, and FAK activity are reversed when miR-204-3p is knocked down. Furthermore, delphinidin reduced DLD-1 cell lung metastasis in a xenograft model (Table 1). Therefore, it might be hypothesized that delphinidin inhibits colorectal cancer metastasis by inhibiting the integrin/FAK signaling pathway, with miR-204-3p playing a pivotal role following delphinidin supplementation [50].

Several immune checkpoints have been discovered and investigated in cancer research in recent decades, including programmed cell death protein 1 (PD-1), lymphocyte-activation gene 3 , cytotoxic T-lymphocyte-associated antigen 4, T cell immunoglobulin and mucin-domain containing-3 , B- and T-lymphocyte attenuator , and the T cell immunoglobulin and immunoreceptor tyrosine-based inhibitory motif domain [51]. The most successful immune checkpoint blockade therapy is anti-PD-1/PD-L1, which has been approved to treat various cancers, including that of the lung, blood, skin, bladder, liver, and kidney [52]. Previous research has looked into the effects of anthocyanin-rich extracts on immunological checkpoints in human colorectal cells [53,54]. By lowering the binding of programmed death-ligand 1 (PD-L1) to PD-1, D3G and its metabolites (delphinidin chloride and gallic acid) have been found to decrease T cell activation in tumor microenvironments. This study shows for the first time that pure anthocyanidins and their metabolites can suppress immune checkpoints [55]. Though anthocyanins are commonly considered as antioxidants, researchers discovered that some anthocyanidins (delphinidin and cyanidin) cause oxidative-stress-related cytotoxicity in metastatic drug-resistant colorectal cancer cells (LoVo and LoVo/ADR) by accumulating ROS, inhibiting glutathione reductase, and depleting glutathione. This implies that anthocyanidins might be utilized to treat metastatic colon cancer as sensitizing agents [56].

### 4.4. Effect of Delphinidin against Lung Cancer

The antitumor effects of anthocyanins are mediated by modulating the Ras-MAPK and PI3K/Akt signaling pathways, which target RTKs such as EGFR, PDGFR, and VEGF/VEGFR. Tumor angiogenesis is a limiting element in malignant tumor growth and spread [57]. The mechanism of angiogenesis is regulated by many cytokines, with VEGF being the most significant positive regulator. Thus, blocking the angiogenesis receptor VEGFR could substantially prevent tumor metastasis. Delphinidin and cyanidin have previously been shown to suppress VEGF production in vascular smooth muscle cells triggered by PDGF by blocking the p38-MAPK and JNK pathways [58]. In A549 lung cancer cells, delphinidin was discovered to have anti-angiogenic properties. Delphinidin reduces the mRNA expression of CoCl_2_- and epidermal growth factor (EGF)-induced hypoxia-inducible factor-1α (HIF-1α). VEGF expression was inhibited as a result of HIF-1 binding to the hypoxia response element (HRE) promoter. Blocking the ERK and PI3K/Akt/mTOR/p70S6K signaling pathways, which result in angiogenesis and tumor growth inhibition, could be utilized as a possible method (Table 1) [23]. These findings suggest that delphinidin may have a new role in anti-angiogenic activity by suppressing HIF-1α and VEGF production.

Similarly, delphinidin treatment reduces cell growth and suppresses proliferation in non-small-cell lung cancer (NSCLC) cell lines, as seen by decreased levels of pro-proliferative signaling molecules, while having no harmful effects on normal bronchial epithelial cells (NHBE). Delphinidin inhibited the phosphorylation of EGFR and VEGFR2, whereas dose-dependent differences in PI3K/p110/p85, pAKT, pERK1/2, pJNK1/2, pp38, cyclin D1, and proliferating cell nuclear antigen (PCNA) were witnessed after 48 h. Tumor formation, angiogenesis, and cell proliferation indicators are reduced in a dose-dependent manner in xenografted athymic nude mice. Thus, delphinidin treatment reduces tumor growth and cell proliferation by suppressing the PI3K/Akt and MAPK signaling pathways [24].

Autophagy is a bidirectional process. Autophagy has been discovered to play a critical role in various biological processes, including cancer growth and prevention, as well as immunity. Malignant tumors, neurological diseases, diabetes, and myocardial infarction may be linked to autophagy dysregulation [59,60]. Delphinidin stimulates autophagy and has been associated with autophagic cell death in a variety of cancers [61]. For delphinidin-induced autophagy, increased expression of microtubule-associated protein light chain 3 (LC3)-II and ATG5 is necessary, which may be due to the inhibition of the AKT/mTOR pathway, culminating in the activation of AMP-activated kinase (AMPK), which enhances the expression of numerous autophagy-related proteins [62]. In line with this, a recent study [63] discovered that delphinidin treatment amplified the impact of γ-ionizing radiation in NSCLC by inducing autophagy and activating the JNK/MAPK pathway. Delphinidin reduced PI3K, AKT, and mTOR phosphorylation in irradiated NSCLC cells and enhanced autophagy-induced cell-death-associated protein expression. In addition, pre-treatment with delphinidin boosted JNK phosphorylation in irradiated NSCLC cells. These data show that delphinidin acts as a radiation-sensitizer by inducing autophagy and activating the JNK/MAPK pathway in NSCLC cells, enhancing apoptotic cell death (Table 1). This study also revealed that increasing autophagy flux might be a potential therapeutic approach for NSCLC (radiation-resistant) anticancer treatment.

### 4.5. Effect of Delphinidin against Skin Cancer

Ultraviolet (UV) radiation causes rapid skin aging, hyperkeratosis (increased epidermal thickness), and acts as a tumor starter and promotor [64]. Numerous studies have shown flavonoids and anthocyanins to have exceptional antioxidant and UV absorption properties, making them ideal candidates for reducing the harmful effects of UV radiation [65]. One study utilized HaCaT cells and SKH-1 hairless mice as skin carcinogenesis models and UVB (280–320 nm, (15–30 mJ/cm^2^) radiation as the carcinogen. UVB irradiation alters DNA directly by forming cyclobutane pyrimidine dimers and pyrimidine–pyrimidone (6-4) photodimers, as well as indirectly by causing ROS to produce 8-hydroxy-2′-deoxyguanosine (8-OHdG) [64,66]. The authors reported that applying delphinidin topically before or after the treatment suppressed UVB-mediated oxidative stress and reduced DNA damage, sparing the cells from UVB-induced apoptosis. In HaCaT cells, delphinidin enhanced and reversed UVB-induced decreased cell viability [67]. On a similar note, another study [68] found that delphinidin protects HaCaT cells by modulating the mechanical characteristics of UVB (100 mJ/cm^2^)-irradiated HaCaT cells (Table 2). According to direct atomic force microscopy measurement, the stiffness of live cells was restored after delphinidin treatment. The authors speculated that this effect was due to an antioxidative and inhibitory effect on the activation of MMP. Delphinidin has previously been shown to suppress the phosphorylation of MAPK-JNK1/2, MEK-ERK1/2, and mitogen-activated protein kinase kinase (MKK)3/6-p38 in human epidermal fibroblasts induced by UVB radiation by targeting decreased nicotinamide adenine dinucleotide phosphate oxidase [69].

A reduction/oxidation (redox) imbalance influences tumor development pathophysiology, with ROS playing a critical role. AP-1, NF-κB, hypoxia-inducible factor 1α, p53, Nrf2, peroxisome proliferator-activated receptor gamma , and β-catenin/Wnt are all transcription factors that can be activated by oxidative stress [70]. The neoplastic change of cells and inflammation are thought to enhance carcinogenesis. In a study, delphinidin was found to be the most effective inhibitor of AP-1 transactivation by blocking 12-O-tetradecanoylphorbol-13-acetate (TPA, a tumor promoter)-induced ERK and JNK signaling cascades compared to other anthocyanidins. Furthermore, delphinidin synergistically inhibited AP-1 activity with superoxide dismutase SOD. TPA treatment causes the production of superoxide anion in mouse skin epidermal (JB6 P+) cells, which increases AP-1 activation and neoplastic transformation. It is worth noting that the B-orthodihydroxyphenyl ring’s structure is linked to anthocyanidins’ antioxidant action, which leads to anticarcinogenesis [71]. Kang and coworkers discovered that delphinidin could interact with Raf1 or MEK1 in an ATP-dependent non-competitive manner to suppress the expression of AP-1 and NF-κB, as well as the expression of COX-2 and the generation of prostaglandin E2 in JB6 P+ cells treated with TPA. Furthermore, delphinidin may reduce TPA-induced cellular transformation via the Ras/Raf/MEK/ERK pathway by suppressing the phosphorylation levels of MEK and its downstream kinases, ERK, ribosomal protein S6 kinase, and mitogen and stress-activated protein kinase (MSK). Delphinidin also reduced EGF and H-Ras-induced JB6 P+ cell transformation, both involved in Raf/MEK/ERK signaling activation (Table 2) [27]. Similarly, according to a recent study on skin cancer prevention, delphinidin prevents TPA-induced neoplastic cell transformation in JB6 P+ cells. Delphinidin treatment increases the expression of the Nrf2/ARE pathway. The Nrf2 signaling pathway is a key component of the body’s basic defense system against oxidative and electrophilic damage [72]. Anthocyanins activate a common ARE in the upstream region of their antioxidant genes, regulated by multiple transcription factors, including Nrf2 [73]. CpG sites in the Nrf promotor region are demethylated as a result of delphinidin activation of Nrf2/ARE, as evidenced by decreased expression of class I/II histone deacetylases and DNA methyltransferases 1 and 3a (DNMT1/3a) (Table 2) [26]. These findings point to the chemopreventive role of delphinidin against skin cancer.

### 4.6. Effect of Delphinidin against Breast Cancer

Breast cancer is the most common type of cancer in women, and is the world’s leading cause of cancer death in this age group [74]. Based on gene expression profiling, breast cancer molecular types are categorized into three categories: estrogen receptor (ER) status, progesterone receptor (PR) status, and human epidermal growth factor receptor 2 (HER2) status. HR refers to a combined assessment of ER and PR status. HR+/HER2– (luminal A subtype), HR+/HER2+ (luminal B subtype), HR−/HER2+ (HER2-enriched subtype), and ER/PR−/HER2– (triple-negative subtype) are molecular subtypes based on this [75]. In breast cancer cells, hyperactivation of signaling pathways such as phosphoinositide 3-kinase–AKT– (PI3K/AKT/mTOR), MAPK, and hepatocyte growth factor (HGF)/Met (hepatocyte growth factor receptor (HGFR)) increases cell growth, proliferation, survival, and cancer hallmarks [76,77]. Invasive human breast cancer has been associated with high amounts of HGF (mesenchymal-derived cytokine) and Met, making them prospective therapeutic targets for the illness. Syed et al. [77] found that delphinidin could inhibit the HGF-induced phosphorylation and activation of HGFR on the human normal mammary cell line MCF-10 A as well as block HGF-mediated activation of NF-ĸB transcription and decrease phosphorylation of IκB kinase α/β (IKKα/β), IκBα, and STAT3 signaling pathways (Table 2).

HER2 is well-known for its role in the pathophysiology of breast cancer. It has been linked to 20–30% of breast cancer tumors, a greater recurrence rate, and a higher mortality rate [78]. Delphinidin has a high affinity for the HER2 receptor, demonstrated using virtual docking techniques [79]. The influence of delphinidin on established breast cancer cell lines of various molecular subtypes was investigated in vitro to determine anticancer efficacy. Delphinidin inhibited proliferation, blocked anchorage-independent growth, and induced apoptosis in ER-positive, triple-negative, and HER2-overexpressing breast cancer cell lines while causing minimal harm to non-transformed breast epithelial cells. MAPK signaling was reduced in triple-negative cells and ER-negative chemically altered MCF10A cells after treatment with delphinidin. Delphinidin also caused a significant level of apoptosis in HER2-overexpressing cells, which was linked to reduced HER2 and MAPK signaling. However, in combination trials, there has been evidence of a possible antagonism between delphinidin and HER2-directed therapy, raising concerns regarding potential pharmacological antagonism when used with other targeted therapies in HER2-overexpressing breast cancer [5]. These data suggest that delphinidin could be helpful in treating breast cancer, but caution against using it as a supplement to other treatments.

Autophagy, a typical cellular mechanism that represents a prospective therapeutic target in cancer treatment, is controlled by the PI3K/mTOR and AMPK signaling pathways [80,81]. In line with this, a study found that delphinidin can inhibit cell proliferation (IC_50_ MDA-MB-453 (40 μM) and BT474 (100 μM)), increase apoptosis (upregulated caspase-3 and -9) and induce protective autophagy in HER2-positive breast cancer (MDA-MB-453 and BT474) cells by activating and suppressing the AMPK/ forkhead box O (FOXO) 3a and AKT/mTOR signaling pathways, respectively. The effect of delphinidin on autophagy was dose-dependent (Table 2) [62]. In ER-positive MCF-7 cells, delphinidin-3-glucoside generates significant cytotoxicity and reduces cell growth in a dose-dependent manner. On the other hand, delphinidin-3-glucoside did not influence ER expression. The cytotoxicity of delphinidin-3-glucoside may be due to the ortho trihydroxylated moiety in ring B, rather than dihydroxylated substituents [82]. Delphinidin increased apoptosis in MDA-MB-231 breast cancer cells according to another study. Proliferation was reduced and apoptosis was increased as a result of chromatin condensation in a concentration-dependent manner. Delphinidin reduced tumor growth in xenografted mice. These findings suggest that delphinidin has the potential to be developed as a cancer prevention drug due to its growth inhibitory effects and activation of apoptosis in human breast cancer cells [83].

MMP family members also play an essential role in tumor invasion and metastasis [84]. Targeting MMP proteins could be an excellent way to see if a chemical has anti-metastasis or anti-invasion properties. The antiproliferative and antiinvasive effects of delphinidin on MMP-9 expression in MCF-7 human breast carcinoma cells generated by phorbol 12-myristate 13-acetate (PMA, tumor promotor and inducer) have been evaluated by Im and coworkers (Table 2). By decreasing NF-κB through MAPK signaling pathways, delphinidin inhibited MCF-7 cell invasion and suppressed MMP-9 expression. This study describes the anti-metastatic potential of delphinidin [85].

Long non-coding RNA (LncRNA) is emerging as a novel tumorigenesis participant and plays an important role [86]. Further research into non-coding RNA has revealed that HOTAIR (HOX transcript antisense intergenic RNA) promotes breast cancer development. Two recent findings [87,88] are worth highlighting. They looked at the effects of delphinidin and its derivative (delphinidin-3-glucoside) on HOTAIR and showed that both compounds decreased the HOTAIR level. Yang et al. [87] found that delphinidin-3-glucoside inhibits HOTAIR expression by promoting interferon regulatory factor-1 (IRF1) expression, blocking Akt activation, and increasing IRF1 binding to the HOTAIR promoter (Table 2). In cancer stem cells originating from breast cancer, HOTAIR can also physically engage with the promoter region to mute a tumor suppressor microRNA (miR34a) [89]. The HOTAIR work was expanded in this direction by focusing on miR34a. Delphinidin treatment inhibits the β-catenin signaling pathway by downregulating HOTAIR while simultaneously upregulating miR34a (tumor suppressor regulator). According to these findings, delphinidin may inhibit breast carcinogenesis via the HOTAIR/miR34a axis [88]. It is regulated by the IRF1 protein, which binds to the HOTAIR promoter and inhibits its activity (decreasing HOTAIR expression). PI3K/Akt activation decreases IRF1 expression while increasing HOTAIR levels. These studies indicate that delphinidin has an effective cancer preventive effect on breast carcinogenesis, with downregulation of HOTAIR playing a key role.

It is worth noting that, while having an antiproliferative impact, delphinidin has been shown to enhance tumor formation and metastasis in an MT-450 tumor model. As a result, clinical trials are currently underway in breast cancer patients receiving radiation treatment and supplemented with anthocyanin-soluble isolates [90].

For the first time, Wu et al. [91] found that anthocyanins from *Hibiscus sabdariffa* (delphinidin being the most abundant anthocyanidin, 69%) exhibited a decrease in mitochondrial membrane potential and triggered autophagy as well as necrosis in MCF-7 cells rather than programmed cell death via the AMPK signaling pathway (Table 2). The initial findings show that anthocyanins from *Hibiscus sabdariffa* have an anticarcinogenic impact; however, further in vivo research is needed to support the anticarcinogenic activity of roselle anthocyanin extract.

### 4.7. Effect of Delphinidin against Hepatic Cancer

Yeh and Yen [92], in their investigation, reported growth-inhibitory effects of anthocyanidins (delphinidin, cyanidin, peonidin, pelargonidin, and malvidin) on the human hepatoma cell line (HepG2 cells). Delphinidin had the most potent inhibitory effects of all the compounds tested. Delphinidin caused apoptosis, which was marked by internucleosomal DNA fragmentation and a rapid increase in caspase-3 activation. These effects were linked to an increase in the expression of c-Jun and JNK phosphorylation. Delphinidin-induced DNA fragmentation was inhibited by N-acetyl-l-cysteine (NAC) and catalase, implying that oxidative stress was the cause of death signaling. However, Feng et al. [93] found that through inducing cell vacuolization, delphinidin could promote apoptosis and antiproliferation in SMMC7721 (hepatocellular carcinoma) cells (Table 2). Endoplasmic reticulum (ER) stress has been linked to an increase in cellular vacuolization. Delphinidin, on the other hand, enhanced cell vacuolization without inducing ER stress. Delphinidin also lipidated LC3 II, an autophagy signal required for autophagosome formation. Furthermore, in the presence of 3-methyladenine, an autophagy inhibitor, delphinidin has been shown to promote necrosis (different from apoptosis) in hepatocellular carcinoma cells. The authors described that the absence of caspase activation was likely related to the deficit in ATP, which is essential for caspase activation. These findings point towards the complex interplay between apoptosis, necrosis, and autophagy, implying that the many death pathways may overlap and that multiple features may be shown simultaneously (Figure 2).

The invasion and progression of tumors are caused by a biological process known as epithelial–mesenchymal transition (EMT), which is promoted by several growth factors such as heparin-binding growth factor, hepatocyte growth factor, EGF), platelet-derived growth factor, and transforming growth factor β (TGF β) [6,94]. An increasing number of studies have described that EGF also critically regulates the activity of EMT in cancers. Delphinidin suppresses EGF-induced morphological changes from EMT, such as the migration and invasion of hepatocellular carcinoma cells by inhibiting the EGFR/AKT/ERK signaling pathway, showing the anti-metastatic effect of delphinidin (Table 2) [95].

### 4.8. Effect of Delphinidin against Leukemia

Human promyelocytic leukemia cells (HL-60) were utilized as a model to investigate the capacity of anthocyanidins to cause apoptosis in a study [96]. When HL-60 cells were treated with six different anthocyanidins, delphinidin, cyanidin, and petunidin, they demonstrated apoptosis as evidenced by morphological changes and DNA fragmentation, whereas malvidin, peonidin, and pelargonidin did not. However, delphinidin was the most potent inducer that triggered HL-60 cell death in a time- and dose-dependent manner. Delphinidin-induced JNK phosphorylation, caspase-3 activation, and DNA fragmentation were efficiently inhibited by antioxidants such as NAC and catalase. The mechanistic investigation suggests that delphinidin induces apoptosis via an oxidation–JNK signaling pathway (Table 2) [96].

Chang et al. [97] discovered that delphinidin present in *Hibiscus sabdariffa* (HA) activated p38-FasL ligands and the pro-apoptosis protein BH3 interacting domain death agonist (BID) pathway, causing apoptosis of human HL-60 cells in a time- and dose-dependent manner. This study describes that HA extract rich in delphinidin could act as a chemopreventive agent in HL-60 cells. In the same year, Hou and coworkers [98] demonstrated the effect of delphinidin-3-sambubioside (from *Hibiscus sabdariffa*) on HL-60 cells’ elevated ROS level, which triggered the apoptosis of HL-60 cells. An elevated ROS level caused by delphinidin-3-*O*-sambubioside has been reported to cause ΔΨm loss through the activation of mitochondrial permeability transition. This process releases cytochrome c (an apoptogenic protein) into the cytosol [99,100] (Figure 2). The study describes for the first time that delphinidin 3-sambubioside could induce apoptosis in HL-60 cells through a ROS-mediated mitochondrial dysfunction pathway (Table 2).

Similarly, delphinidin is the most prevalent phytoconstituent (69%) in roselle plant extract, which drastically triggers leukemia cell cycle G2/M arrest in HL-60 cells by affecting the activity of the p27/21, Cdc2, and ataxia telangiectasia mutated/checkpoint kinase (1/2)/cell division cycle 25C (ATM-Chk1/2-Cdc25C) signaling pathways independent of p53 [101]. Glyoxalase I (GLO I) is an enzyme that aids tumor cells in metabolizing methylglyoxal, a substance that can trigger apoptosis. In HL-60 cells, delphinidin treatment suppresses (IC_50_ value of 1.9 μM) enzyme activity and promotes apoptosis (Table 2) [102].

Delphinidin-3-*O*-rutinoside and delphinidin-3-*O*-glucoside facilitated the pro-apoptotic impact (governed by caspase-3) of blackcurrant juice on Jurkat human leukemia cells, according to León-González et al. [103]. Jurkat cells were induced to be pro-apoptotic by delphinidin glycosides, but not by cyanidin derivatives. These data suggest that the hydroxyl group at the 5′ position of delphinidin’s B ring is critical for inducing apoptosis in Jurkat cells.

### 4.9. Effect of Delphinidin against Bladder and Mesenchymal Tumors

Bladder cancer is the second most prevalent type of cancer, and its advancement is mainly linked to the development of spontaneous metastases, which further aggravate the disease [104]. The disease is a severe problem, and despite great medical discoveries over the last 15 years, the death rate from bladder cancer has only decreased by 5% [105]. Delphinidin has an anticancer effect on the human urinary bladder cancer cell line T24, as evidenced by increased cytotoxicity, the dose-dependent death of T24 cells (as measured by annexin V/propidium iodide flow cytometry), and an increase in ROS production, according to Kang et al. (Table 2) [20]. This research suggests that delphinidin may be a promising treatment option for urinary bladder cancer in the future.

Filipiak et al. [106] discovered that delphinidin-3-*O*-glucoside decreased HT1080 (human fibrosarcoma cell line) cell viability, whereas cyanidin-3-*O*-glucoside had no effect. Delphinidin-3-*O*-glucoside and its metabolite, gallic acid, have been discovered to cause cytotoxicity in HT1080 cells by inhibiting MMP-2/-9 activity (Table 2).

### 4.10. Effect of Delphinidin against Glioma

The most frequent type of malignant and invasive primary brain tumor is glioma. Despite significant advancements in glioma detection and treatment in recent decades, the prognosis remains poor. MicroRNAs have sparked a revolution in molecular biology in recent decades, and they have been recognized as crucial players in malignancies. According to research, dysregulated miRNAs can rewire several critical cellular and molecular processes intimately implicated in glioma’s beginning and progression [107,108]. MicroRNA-137 (miR-137), widely expressed in the brain, has powerful inhibitory effects on the development, metastasis, and invasion of glioma cells [107,109]. In glioblastoma, miR-137 expression is epigenetically suppressed, and overexpression of miR-137 inhibits cancer cell invasion, showing that miR-137 is a tumor-suppressive miRNA [110]. Overexpression of miR-137 enhances delphinidin-mediated apoptosis by activating both intrinsic and extrinsic mechanisms. Furthermore, combining delphinidin and miR-137 inhibits a number of cellular components involved in cellular survival, proliferation, invasion, and angiogenesis [110]. Thus, treating glioblastoma cells with delphinidin and overexpressed miR-137 could be a viable therapeutic method. As mentioned above, many growth factors induce EMT. TGF-β has been shown to operate as both a tumor suppressor and a tumor promoter during carcinogenesis [111]. Considered the most potent inducer of EMT, activating type I and II serine–threonine kinase receptors, leading to the activation of receptor-regulated Smads (R-Smads). R-Smads translocate to the nucleus and interact with different transcription factors (Snail, Slug, and Twist) to control EMT target genes[112]. Besides the Smad signaling pathway, TGF-β also activates p38 MAP kinases, JNK, and ERK (examples of non-Smad signaling) [113]. Inhibiting EMT via TGF signaling pathways may be effective in halting tumor progression. Delphinidin is a potent EMT inhibitor that inhibits cell migration in human U-87 MG glioblastoma, as evidenced by reduced TGF/Smad2 and TGF/ERK signaling pathways, as well as decreased expression of EMT markers, fibronectin and Snail, among all anthocyanidins studied (Table 2) [114].

### 4.11. Effect of Delphinidin against Osteosarcoma (OS)

The most common tumor that causes cancer-related mortality in children is OS. Delphinidin treatment induces apoptosis and inhibits EMT-related protein expression in human OS cell lines through ERK/p38 MAPK signaling [21]. In another study, delphinidin administration led to dose-dependent reductions in OS cell viability of 96 percent, 55 percent, 32 percent, and 22 percent at 10, 50, 100, and 200 μg/mL, respectively. Furthermore, no reduction in viability was seen after pretreatment with the antioxidant NAC. The study indicates that delphinidin-induced cell death is directly linked to ROS formation, and that delphinidin operates as an anticancer agent, regardless of its anti-oxidant capacity (Table 2) [115].

## 5. Concluding Remarks, Challenges and Future Perspectives

Based on the presented data, delphinidin and its glycosides appear to be a promising chemopreventive measure in prostate, breast, and ovarian cancers, as well as lung, colorectal, skin, liver, and other cancer types. Delphinidin and its glycosides, as well as combinations with other anthocyanidins, hydrolysis, and conversion to gallic acid, as well as other products, appear to protect against the development of cancer. Moreover, recent studies have proposed that the inhibitory mechanisms of delphinidin on cancer chemoprevention can be categorized as (1) anti-invasive potential, (2) anti-angiogenesis, (3) the molecular mechanism involved in apoptosis induction, (4) blockage of insensitive signaling pathways, and (5) regulation of the expression of tumor suppression genes. Furthermore, delphinidin reduced cancer cell invasive capacity by inhibiting the Wnt/β-catenin pathway or by downregulating the EGFR/AKT/ERK, TGF/Smad2 and TGF/ERK signaling pathways linked with EMT decrease. The downregulation of MMPs and overexpression of their inhibitors indicated delphinidin’s ability to reduce the migratory potential of tumors. Delphinidin has been shown to influence numerous signaling pathways such as JNK/MAPK and AKT/mTOR, lowering their activity and reducing cancer cell survival and proliferation by inducing cell death via apoptotic, autophagic, or necrotic mechanisms. The anti-angiogenesis effects of delphinidin are mediated by targeting RTKs (EGFR and VEGFR) by acting on Ras-MAPK and PI3K/Akt signal cascade pathways. Initially, delphinidin suppresses inflammation by acting on the PI3K/Akt and NF-κB pathways to limit COX2 and iNOS, preventing normal cells from being transformed by controlling the expression of phase II antioxidant enzymes to accomplish antioxidation via the Nrf2/ARE signal system. During the development stage, delphinidin causes cancer cells to apoptose by activating the caspase system, mediated by ROS and JNK/p38MAPK. Furthermore, delphinidin can enhance the sensitization of cancer cells for anticancer compounds for improving chemotherapy sensitivity. Innovative ideas for customized cancer treatment are rationalized by a better grasp of the latest updates on inhibitors, antagonists, and activators of diverse signaling pathways.

D3G and its metabolic forms, including delphinidin and gallic acid, have been shown to induce apoptosis [55]. D3G and its metabolites appear to play a key role in the anti-carcinogenic activity of the plant extracts. Despite its promising function in cancer prevention and treatment, delphinidin has low bioavailability when delivered as a pure, active compound, posing a significant barrier to widespread usage. Other delivery routes should be created with state-of-the-art approaches, such as nanotechnology, encapsulation, and specialized targeting, to dodge delphinidin metabolism after oral ingestion and therefore boost its bioavailability. However, by combining these compounds with other phytochemicals, anticancer medicines, or delphinidin-loaded nanotechnology-based delivery methods [116], the bioavailability and, hence, the efficacy of delphinidin can be increased. Nano-formulations include gold nanoparticles, polymeric nanoparticles, nanosuspensions, and liposomes [117]. Nano-formulations of natural phytoconstituents improve the bioavailability, stability, and efficacy of chemoprevention and treatment, which rely on dosages [118]. Nevertheless, toxicological studies are required for these nano-formulations, as they open a new chapter in oncologic treatment.

Concerning food industry applications, delphinidin is susceptible to degradation. Its stability is affected by pH, solvent type, temperature, concentration, and presence of light and oxygen. The best strategy could be the use of nano/microencapsulation methods [119]. A recent study pointed out the high pH (3.0–9.0) stability of pyranoanthocyanidins (new derivative, found in fermented fruit juices and aged red wine) and substantial anticancer potential of anthocyanidins compared to anthocyanin [120]. Future research may be focused on the anticancer potential of pyranoanthocyanidins. The majority of the papers included in this review are from in vitro investigations. Moreover, according to Xiao and Hogger’s [121] investigation, polyphenol stability is higher in plasma than in culture conditions, therefore there may be a discrepancy between in vitro and in vivo results. To add to our surprise, Thiele and colleagues [90] found that delphinidin treatment promoted tumor growth and metastasis in an MT-450 tumor model (syngeneic experimental rats), implying that the antiproliferative effect of delphinidin in cultured cells does not ineludibly reproduce the tumor response to this anthocyanidin in vivo. However, delphinidin therapy in human HT29 colon and rat MT-450 mammary cancer cells inhibited growth and caused apoptosis in vitro. In vivo, it also inhibited angiogenesis and tumor-induced lymphangiogenesis. As a result, future research should concentrate on the importance of in vitro results translating into in vivo experiments. Despite this, evidence from human studies, particularly in cancer-related areas, is still lacking. Few recent studies have evaluated the efficacy of Delphinol^®^ clinically. For example, Delphinol^®^ (180 mg) improved oxidative stress in overweight volunteer smokers and healthy adults [122]. In another study, Delphinol^®^ (180 mg) lowered blood glucose level significantly and improved the blood lipid profile of prediabetic subjects [123]. A recent clinical study depicted the role of Delphinol^®^ in maintaining healthy skin conditions [124]. Thus, clinical testing of delphinidin’s anticancer potential is required for a reliable conclusion. Furthermore, combining anthocyanin and probiotics as a novel cancer treatment technique should be pursued in the future. The use of anthocyanin and activated gut flora as efficient immunological checkpoint inhibitors against mice colon cancer was recently established [125]. Changes in inflammatory status and methylation of the *SFRP2* (secreted frizzled-related protein 2) gene by anthocyanin through modulated gut microbiota composition, are thought to play a role in colorectal cancer chemoprevention [126].

## Figures and Tables

**Table 1:** Preventive and anticancer effects of delphinidin and its glycosides on models of prostate, ovary, colorectal, and lung cancer.

Ref.	Type	Cell/Animal	Intervention	Major Findings
**Prostate Cancer**				
[34]	In vitro	PC-3	Del (30, 60, 90, 120, and 180 μmol/L), 48 h	↓ Cell viability IC_50_ = 90 μmol/L, ↑ apoptosis, ↑ caspase-3, ↑ caspase-9, induction of PARP cleavage, ↑ BAX/Bcl-2 ratio, dose-dependent cell cycle arrest (G2/M phase), ↓ cyclin D1, ↓ cyclin A, ↓ cdk1, ↓ cdk2, and inhibited translocation as well as DNA binding of NF-κB and pIKKγ protein
	In vivo	Athymic (nu/nu) male nude mice	Del (2 mg/animal) in 1:10 DMSO and normal saline, 3 times/week	No toxicity, inhibited tumorigenicity, ↓ Bcl-2 protein, ↑ BAX, ↓ cyclin D1 and NF-κB, and inhibited proliferation marker (Ki67 and PCNA) expression
[25]	In vitro	PC-3	Del (15, 30, 60, 120, 180, and 240 μM), 72 h	↓ β-catenin and its target proteins—Axin2, LEF1, cyclin D1, c-myc, and TCF1—↑ E-cadherin
[32]	In vitro	LNCaP	Del (30, 60, and 90 μM) TRAIL (0, 50, 100, and 150 ng/mL), 12 h	Dose-dependent antiproliferative effect, altered cell and nucleus morphology, ↑ cleaved PARP, ↑ caspase-8, ↑ caspase-9, ↑ cleaved caspase-3, ↑ caspase-7, ↑ DR5, ↑ p21, ↑ BAX, ↓ Bcl-2, ↓ XIAP, ↓ MCL-1, ↓ cIAP-2, ↓ survival, and ↓HDAC3
[29]	In vitro	LNCaP	Del (50, 100, and 150 μM), 24 h	↑ Apoptosis, ↓ PARP-1, ↓HDAC3, ↑BAX, ↑PUMA, ↑p21, and ↑ p53 acetylation
[39]	In vitro	LNCaP	Del-3-glu (3, 10, 30, and 100 μM), 48 h	↓ DHT, ↓AR, ↓PSA, and ↓SRD5A1
**Ovary Cancer**				
[42]	In vitro	SKOV3	BDNF (100 nM), 24 h Del (50 and 75 µΜ), 24 h	Dose-dependent inhibition of migration ability, ↓ MMP-2, ↓ MMP-9, and inhibited p-Akt and NF- κB translocation
[22]	In vitro	SKOV3	Del (0.1, 1, and 10 µM), 48 h	↓ p-Akt, ↓ pP70S6K, ↓ pS6, ↓ p-ERK1/2 MAPK, and ↓ p-P38 MAPK
[44]	In vitro	ES2	Del (0.1, 1, 10, 50, and 100 µM), 48 h	↓ Proliferation, ↓ metastasis, ↓ PI3K/Akt, and ↓ ERK1/2/JNK
**Colorectal Cancer**				
[46]	In vitro	HT-29	H_2_O_2_ (50 μM) + Del 25 μg/mL, 24 h	↓ PGK1 activity
[47]	In vitro	HCT-116	Del (30, 60, 120, 180, and 240 μM), 48 h	Dose-dependent inhibition of viability, IC_50_ = 110 μM, ↑ apoptosis, ↑ cleaved PARP, ↓ procaspase-3, ↓ procaspase-8, ↓ procaspase-9, ↓ Bcl-2, ↑ BAX, ↑ G2/M phase arrest, ↑ p53, ↑ p21WAF1/Cip1, and ↓ NF-κB
[48]	In vitro	HCT-116	Del (80, 100, and 120 μM), 48 h	Dose-dependent inhibition of viability, IC_50_ = 106 μM, ↑ BAX, ↑ caspase- 3, ↑ caspase-8, ↑ caspase- 9, ↑ cytochrome C, ↓ p-STAT-3, ↓ p-p38, and ↓ p-ERK1/2
**Colorectal Cancer**				
[50]	In vitro	DLD-1, SW480, and SW620	Del (20, 40, 60, 80, and 100 μM), 24 h	↓ Migration, ↓ invasion, ↓ integrin αV/β3, ↓ FAK/Src/paxillin signaling, ↓ Snail, ↓ Twist, ↓ Slug, ↓ β-catenin, ↓ MMP-2, ↓ EMT, and ↑ E-cadherin
	In vivo	Balb/c nude mice	DLD-1 implantation + Del 100 μM, 24 h	Reduced metastasis No change in liver weight
[55]	In vitro	CRC cells (HCT-116, HT29); PBMCs pre-treated with anthocyanins and co-cultured with HCT-116 and HT-29	D3G, cyanidin-3-O-glucoside and its metabolites (delphinidin chloride and GA) (100–600 μg/mL)	↓ PD-L1 expression, ↓ PD-1 expression, and ↓ binding of PD-L1 to PD-1
**Lung Cancer**				
[23]	In vitro	A549	CoCl_2_ (200 µM) and EGF (20 ng/mL), Del (10, 20, and 40 µM), 24 h	↓ Angiogenesis, ↓ HIF-1α, ↓VEGF, and ↓ PI3K/Akt/mTOR/p70S6K
[24]	In vitro	NSCLC	Del (5, 10, 20, 40, and 60 μM), 3 h	↑ Caspase-3/9 and ↓ anti-apoptotic proteins (Bcl-2, Bcl-xL, and Mcl-1), ↑ Pro-apoptotic proteins (BAX and BAK) and ↓ EGFR as well as VEGFR2
	In vivo	Female athymic (nu/nu) nude mice	Del (1, 2 mg/animal) three times/week	Inhibited tumor growth, ↓ Ki67, ↓ PCNA, ↓CD31, ↓ VEGF, and apoptosis induction
[63]	In vitro	NSCLC	Del (1, 5, 10, and 20 μM), 24 h + γ ray irradiation	↑ Apoptosis, ↑ JNK/MAPK pathway, ↓ mTOR pathway, ↑ LC3-II, ↑ ATG5, and ↑ ATG12

Del: delphinidin; PARP: poly(ADP-ribose) polymerase; Bcl-2: B-cell lymphoma 2; BAX: Bcl-2-associated X protein; CDK1/2: cyclin-dependent kinase 1/2; NF-κB: nuclear factor-kappa B; IKK-γ: inhibitor of nuclear factor-kappa B kinase subunit gamma; PCNA: proliferating cell nuclear antigen; TCF1: T-cell factor 1; LEF1: lymphoid enhancer factor 1; DR5: death receptor 5; XIAP: X-linked inhibitor of apoptosis protein; MCL-1: myeloid cell leukemia 1; cIAP-2: cellular inhibitor of apoptosis; HDAC3: histone deacetylase 3; PUMA: p53 upregulated modulator of apoptosis; DHT: dihydrotestosterone; AR: androgen receptor; PSA: prostate-specific antigen; SRD5A1: steroid 5α-reductase type I; MMP-2/9: matrix metalloproteinases 2/9; p-Akt: phosphorylated Akt; p70S6K: phosphorylated 70S6 kinase; pS6: phosphorylated S6; ERK1/2: extracellular signal-regulated protein kinase 1/2; MAPK: mitogen-activated protein kinase; PI3K: phosphatidylinositol-3-kinase; JNK: c-Jun N-terminal kinase; PGK 1: phosphoglycerate kinase 1; pSTAT3: phosphorylated signal transducer and activator of transcription 3; FAK: focal adhesion kinase; Src: steroid receptor coactivator; EMT: epithelial–mesenchymal transition; PD-L1: programmed death-ligand 1; PD-1: programmed cell death protein 1; ROS: reactive oxygen species; LC3-II: microtubule-associated protein light chain 3-II; HIFα: hypoxia-inducible factor alpha; NSCLC: non-small-cell lung cancer; VEGF: vascular endothelial growth factor; EGFR: epidermal growth factor receptor; VEGFR2: vascular endothelial growth factor receptor 2; CD31: cluster of differentiation 31; mTOR: mammalian target of rapamycin; ATG-5/12: autophagy-related gene 5/12; D3G: delphinidin-3-*O*-glucoside; and C3G: cyanidin-3-*O*-glucoside.

**Table 2:** Preventive and anticancer effects of delphinidin and its glycosides in models of skin cancer, breast cancer, hepatic cancer, leukemia, bladder and mesenchymal tumors, glioma, and osteosarcoma.

Ref.	Type	Cell/Animal	Intervention	Major Findings
**Skin Cancer**				
[67]	In vitro	HaCaT	Pretreatment Del (1, 5, 10, 15, and 20 μM), 24 h, and UVB (15–30 mJ/cm^2^), 24 h	↑ Cell viability, ↓ apoptosis, ↓ cleaved PARP, ↓ lipid peroxidation, ↓ PCNA degradation, ↓ BAX, ↑ Bcl-2, and inhibited ↓ procaspase-3, -8, and -9
	In vivo	SKH-1 hairless mouse skin	Del 1 mg/0.1 mL DMSO and UVB (180 mJ/cm^2^)	↓ CPDs, ↓ 8-OHdG, and ↓ apoptotic cells
[68]	In vitro	HaCaT	Del (5 and 10 μM) and UVB (100 mJ/cm^2^)	↑ Elastic modulus
[27]	In vitro	JB6 P+	Del (5, 10, 20, and 40 μM), 30 min+ TPA 4 h	Del (10 μM) inhibited 43% neoplastic transformation, ↓ COX-2, ↓PGE2, ↓ AP-1, ↓ NF-κB, ↓ p-ERK, ↓ p-90RSK, ↓ p-MSK, suppressed Raf1 and MEK1 activities
[26]	In vitro	JB6 P+	Del (10, 20, 40, 60, 80, and 100 μM), 5 days + TPA10 ng/mL	↑ Nrf2, ↑ Hmox1, ↑ Nqo1, ↑ SOD1, ↓ CpG methylation, ↓ DNMTs, and ↓ HDACs
**Breast Cancer**				
[77]	In vitro	MCF-10A	Del (5, 10, 20, and 40 μM), 3 h + HGF (40 ng/mL), 30 min	↓ Cell viability, suppressed p-Met, ↓ p-FAK, ↓ p-src, ↓ p-Crk, ↓ p-JNK, ↓ p-SHP-2, ↓ p-Gab1, and ↓ GRB2, inhibited Ras-ERK MAPK and PI3K/AKT NF-κB/p65 pathways
[5]	In vitro	HCC1806, MDA231, MDA468, SKBR3, MDA453, BT474, and MCF7	Del (12.5, 25, 50, and 100 µg/mL), 6 days; effective at 50 µg/mL, 48 h	↑ Apoptosis, blocked anchorage independent growth and migration, ↓ p-HER2, ↓ p-Akt, and ↓ p-ERK1/2
[62]	In vitro	MDA-MB-453 and BT474	Del (20, 40, 80 µM), 48 h	↓ Proliferation, ↑ cleaved caspase-9 and -3, ↑ LC3-II/LC3-I, ↑ Atg5-Atg12, inhibited mTOR signaling, and activated AMPK signaling
[82]	In vitro	MCF-7	Delphinidin-3-glucoside (12.5, 25, 50, and 100 µM), 48 h	↑ Antiproliferative effect
[83]	In vitro	MDA-MB-231	Del (12.5, 25, and 50 µM), 24 h	↓ Proliferation concentration-dependent manner, ↑ p53, ↓ Bcl-2, and ↓ p-GSK3β
	In vivo	Female nude mice	10 mg/kg	↑ Apoptosis
[85]	In vitro	MCF-7	Del (15, 30, 60, and 90 µM) + PMA (100 nM), 24 h	↓ Invasion and migration, ↓ MMP-9, ↓ p-p38, ↓ p-JNK, inhibited translocation of p65, and ↑ IκBα
**Breast Cancer**				
[87]	In vitro	MCF10A	Del (10, 20, and 40 µM), 24 h	↓ HOTAIR expression, ↓ Akt, and ↑ IRF1
	In vivo	Xenografted female BALB/c athymic mice	Del (40 mg/kg/day)	Inhibited breast tumor growth and ↓ HOTAIR expression
[88]	In vitro	MDA-MB-231, MCF-7, and MDA-MB-453	40 μM, 24 h	↓ HOTAIR expression, ↑ miR-34a, ↓ β-catenin, ↓ Gsk3β, ↓ c-Myc, ↓ cyclin-D1, and ↓ MMP-7
	In vivo	MNU-induced female SD rats	100 mg/kg/rat/day	Lower (43.7%) cancer incidence, ↓ proliferation, and no adverse effect
[91]	In vitro	MCF-7	HA (0, 1, 2, and 3 mg/mL), 24 h	↑ LC3-II, ↑ p-AMPK, and ↓ mitochondrial membrane potential
**Hepatic Cancer**				
[92]	In vitro	HepG2	Del (50, 100, 150, and 200 μM), 24 h	↑ BAX, ↓ Bcl-2, ↑ DNA fragmentation, ↑ LDH leakage, ↑ c-Jun, ↑ p-JNK, and ↑ intracellular ROS
[93]	In vitro	SMMC7721	Del (100 and 150 μM), 24 h	↑ LC3 lipidation and ↑ cellular vacuolization
[95]	In vitro	Huh7 and PLC/PRF/5	Del (30, 40, 80, and 100 μM), 24 h or 48 h	EGF-induced ↓ EMT, ↓ EGFR, ↓ MMP2, ↓ ERK, ↓AKT, ↑ E-cadherin, ↓ vimentin, ↓ Snail, ↓ MMP-2, and ↓ EGFR/AKT/ERK
**Leukemia**				
[96]	In vitro	HL-60	Del and anthocyanidins (100 μM), 6 h	↑ c-Jun, ↑ p-JNK, ↑ cleaved caspase-3, and ↑ apoptosis
[97]	In vitro	HL-60	Del (3 mg/mL), 24 h	↑ Apoptosis, ↑ p-p38, ↑ p-c-jun, ↑ cleaved caspase-8, ↑ cleaved caspase-3, ↑ cytochrome C (in cytosol), ↑ Fas, and ↑ FasL
[98]	In vitro	HL-60	Delphinidin-3-sambubioside (25, 50, 75, 100, and 125 μM), 6 h	↑ DNA fragmentation, ↑ apoptosis, ↑ activation of caspase-9, -8, and -3, ↑ cytochrome C (in cytosol), ↓ BID, and loss of mitochondrial membrane potential
[101]	In vitro	HL-60	HA extract (69% delphinidin) (0, 0.1, 0.3, 0.5, and 0.7 mg/mL), 24 h	Cell cycle arrest at the G2/M phase and activation of ATM/cellular checkpoint kinase pathway
[102]	In vitro	HL-60	Del (10, 30, 100 μM), 24 h	Inhibited GLO I (IC_50_ = 1.9 μM) and ↑ apoptosis
[103]	In vitro	Jurkat	Delphinidin-3-O-glucoside and delphinidin-3-O-rutinoside (100 μM), 24 h	↑ ROS generation, ↓ p-Akt, ↓ p-Bad, ↓ Bcl-2, and ↓ UHRF
**Bladder and Mesenchymal Tumors**				
[20]	In vitro	T24	Del (10, 20, 30, 40, 50 and 60 μg/mL), 24 h	↓ Proliferation, ↑ apoptosis, and ↑ ROS generation
[106]	In vitro	HT1080	Del-3-glu (10 and 100 μM), 36 h	Inhibits MMP-2 (IC_50_ = 16.0 μM) and MMP-9 (IC_50_ = 13.6 μM)
**Glioma**				
[110]	In vitro	U87MG and LN18	DPN (10, 25, and 50 μM), 24 h	↓ Viability, ↓ p-Akt, ↓ NF-κB, ↓ VEGF, ↓ b-FGF, ↓ EGFR, ↓ MMP-9, ↓ MMP-2, ↑ caspase-8, ↑ truncated BID, ↑ BAX, ↑ caspase-3, ↑ caspase-6, and ↓ Bcl-2
[114]	In vitro	U-87 MG	Del (35 and 50 μM), 24 h	↓ Migration, ↓ TGFβ/Smad2, ↓ TGFβ/ERK, ↓ fibronectin, and ↓ Snail
**Osteosarcoma**				
[21]	In vitro	HOS and U2OS human osteosarcoma	Del (10, 25, 50, 75, and 100 µM), 24 h	↑ apoptosis, ↑ E-cadherin, ↓ N-cadherin, ↓ Snail, ↓ Slug, ↓ EMT, ↓ ERK, and P38 phosphorylation
[115]	In vitro	U2OS	Del (10, 50, 100, and 200 μM), 48 h	↓ Cell viability, ↑ ROS, ↑ LC3-II, ↑ autophagosome formation, and ↓ p62

Del: delphinidin; PARP: poly(ADP-ribose) polymerase; PCNA: proliferating cell nuclear antigen; Bcl-2: B-cell lymphoma 2; BAX: Bcl-2-associated X protein; DMSO: dimethyl sulfoxide; CPD: cyclobutane pyrimidine dimer; 8-OHdG: 8-hydroxy-2′-deoxyguanosine; UVB: ultraviolet B; TPA: 12-O-tetradecanoylphorbol-13-acetate; COX-2: cyclooxygenase 2; PGE2: prostaglandin E2; AP-1: activator protein 1; NF-κB: nuclear factor-kappa B; p-ERK: phosphorylated extracellular signal-regulated protein kinase; p-p90RSK: phosphorylated p90 ribosomal S6 kinase; p-MSK: phopshorylated mitogen- and stress-activated protein kinase; MEK: mitogen-activated protein kinase kinase; Nrf2: nuclear-factor-E2-related factor 2; Hmox1: heme oxygenase 1; Nqo1: NAD(P)H/quinone oxidoreductase 1; SOD: superoxide dismutase; DNMT: DNA methyltransferase; HDACs: histone deacetylases; p-FAK: phosphorylated focal adhesion kinase; p-JNK: phosphorylated c-Jun N-terminal kinase; p-SHP-2: phosphorylated SH2 domain-containing protein tyrosine phosphatase-2; HGF: hepatocyte growth factor; Grb2: growth factor receptor-bound protein 2; p-Gab1: Grb2-associated binder 1; MAPK: mitogen-activated protein kinase; PI3K: phosphatidylinositol-3-kinase; p-HER2: phosphorylated human epidermal growth factor receptor 2; LC3-II/I: microtubule-associated protein light chain 3-II/I; ATG-5/12: autophagy-related gene 5/12; mTOR: mammalian target of rapamycin; AMPK: 5’ AMP-activated protein kinase; p-GSK3β: phosphorylated glycogen synthase kinase-3β; MMP-9/2: matrix metalloproteinase-9/2; IκBα: inhibitor of nuclear factor (NF)-κB α isoform; PMA: phorbol 12-myristate 13-acetate; HOTAIR: HOX transcript antisense RNA; IRF1: interferon regulatory factor 1; SD: Sprague Dawley; p-AMPK: phosphorylated 5’ AMP-activated protein kinase; MNU: 1-methyl-1-nitrosourea; LDH: lactate dehydrogenase; ROS: reactive oxygen species; EMT: epithelial–mesenchymal transition; EGF: epidermal growth factor; EGFR: epidermal growth factor receptor; FasL: Fas ligand; BID: BH3 interacting domain death agonist; GLO I: glyoxalase I; IC_50_: the half-maximal inhibitory concentration; p-Bad: phosphorylated Bcl-2-associated death promoter; p-Akt: phosphorylated Akt; UHRF1: ubiquitin-like PHD ring finger 1; VEGF: vascular endothelial growth; and b-FGF: basic fibroblast growth factor.

## Data Availability

Not applicable.
